# Plasma metabolomics profiling of maintenance hemodialysis based on capillary electrophoresis - time of flight mass spectrometry

**DOI:** 10.1038/s41598-017-08327-w

**Published:** 2017-08-15

**Authors:** Shuxin Liu, Lichao Wang, Chunxiu Hu, Xin Huang, Hong Liu, Qiuhui Xuan, Xiaohui Lin, Xiaojun Peng, Xin Lu, Ming Chang, Guowang Xu

**Affiliations:** 1 Nephrology Department, Dalian Municipal Central Hospital, 826 Xinan Road, Dalian, 116033 China; 2 CAS Key Laboratory of Separation Sciences for Analytical Chemistry, Dalian Institute of Chemical Physics, Chinese Academy of Sciences, 457 Zhongshan Road, Dalian, 116023 China; 3 State Key Laboratory of Fine Chemicals, Dalian University of Technology, Dalian, 116024 China; 4 School of Computer Science & Technology, Dalian University of Technology, 116024 Dalian, China; 5 University of Chinese Academy of Sciences, Beijing, 100049 China

## Abstract

Uremia has been a rapidly increasing health problem in China. Hemodialysis (HD) is the main renal replacement therapy for uremia. The results of large-scale clinical trials have shown that the HD pattern is crucial for long-term prognosis of maintenance hemodialysis (MHD) in uremic patients. Plasma metabolism is very important for revealing the biological insights linked to the therapeutic effects of the HD pattern on uremia. Alteration of plasma metabolites in uremic patients in response to HD therapy has been reported. However, HD-pattern-dependent changes in plasma metabolites remain poorly understood. To this end, a capillary electrophoresis-time of flight mass spectrometry (CE-TOF/MS)-based metabolomics method was performed to systemically study the differences between HD and high flux hemodialysis (HFD) on plasma metabolite changes in patients. Three hundred and one plasma samples from three independent human cohorts (i.e., healthy controls, patients with pre-HD/post-HD, and patients with pre-HFD/post-HFD) were used in this study. Metabolites significantly changed (*p* < 0.05) after a single HD or HFD process. However, 11 uremic retention solutes could be more efficiently removed by HFD. Our findings indicate that a CE-TOF/MS-based metabolomics approach is promising for providing novel insights into understanding the effects of different dialysis methods on metabolite alterations of uremia.

## Introduction

Chronic kidney disease (CKD) is a worldwide health problem in a rapidly increasing population and can develop into end-stage renal disease (ESRD)^1^. Hemodialysis (HD) is a useful and common treatment for uremia. A large-scale analysis of clinical outcomes has shown that HD pattern is crucial for long-term prognosis of uremic patients receiving maintenance hemodialysis (MHD). Investigating plasma metabolic changes in uremic patients treated with different dialysis patterns is a promising approach to understand uremic pathological alterations and the effects of different dialysis modes on uremia and even to provide valuable information for future personalized HD therapy. To date, only a small number of special uremic toxins have been studied with regard to their clearance during a single dialysis process^2, 3^. Eleftheriadis *et al*. reported abnormal activities of some enzymes in HD patients^4^. Blacher *et al*. and Kalim *et al*. investigated complex complications in ESRD^5, 6^. Although much attention has been paid to ESRD and dialysis from different aspects, the particular alterations of small molecular hydrophilic metabolites in uremic patients after one course of different types of dialysis (e.g., HD and high flux hemodialysis (HFD)) still have rarely been studied. Metabolomics is a field in which small molecule compounds that play crucial roles in physiological and pathological processes associated with health and disease statuses are comprehensively studied by analyzing biological samples^7^. It has been a powerful tool for investigating various diseases^8^. Pawlak *et al*. reported that the kynurenine pathway had a close relationship to pathogenesis of arterial damage in ESRD, and kynurenine and quinolinic acid were novel factors linked to atherosclerosis^9^. To differentiate CKD stages, Shah *et al*. investigated plasma metabolites in 4 stages of CKD by gas and liquid chromatography coupled to mass spectrometry^10^. They discovered several biomarkers to effectively discriminate stages of CKD. Our previous research also showed significant lipid alterations in uremia patients by liquid chromatography coupled with mass spectrometry^11^. Currently, metabolomics has been a sophisticated and important platform for studying kidney diseases.

In this study, a capillary electrophoresis-time of flight mass spectrometry (CE-TOF/MS)-based metabolomics approach was used to define small molecular hydrophilic metabolites in plasma from different uremic patients before (pre-) and after (post-) HD and HFD (e.g., pre-HD, post-HD, pre-HFD, post-HFD) and compare those results to healthy controls. Multivariate and univariate statistical analyses were carried out to analyze dialysis-pattern dependent changes in plasma metabolites to acquire a better understanding of the pathogenetic information of uremia and to investigate the differences between HD and HFD on uremia. Specifically, Support Vector Machines-Recursive Feature Elimination (SVM-RFE)^12^ was used for statistical analysis of the current metabolomics data.

## Results

### Clinical parameters

To acquire reliable results, a 1:2 propensity score matching method was used to select suitable volunteers in this experiment. Finally, a total of 42 stable HFD patients (male/female: 26/16), 85 stable HD patients (male/female: 57/28) and 47 healthy controls (male/female: 26/21) were enrolled. There was no significant differences in age among three groups. The mean age was 50.88 ± 10.59 years in HFD patients, 48.96 ± 12.85 years in HD patients and 51.55 ± 10.85 years for the controls, respectively. The baseline clinical parameters of HFD and HD patients are shown in Table 1. No significant differences existed between the HFD group and the HD group including age, gender, primary disease, dialysis age, hemoglobin, albumin, Glutamic-pyruvic transaminase, pre/post-dialysis blood urea nitrogen (BUN) and pre/post-dialysis serum creatinine (Scr), Kt/V, pre-dialysis serum potassium, sodium, calcium, and phosphorus. These results showed that the basic clinical characteristics between the two groups were comparable.

**Table 1:** Basic clinical characteristics of the samples in the research set.

	HFD	HD	*p*
No.	42	85	
Age	50.88 ± 10.59	48.92 ± 12.82	0.39
Gender (male/female)	26/16	57/28	0.69
Primary disease			0.71
Chronic glomerulonephritis	26	47	
Diabetic kidney disease	5	8	
Hypertension	9	22	
others	2	8	
Dialysis age (month)	82.2 ± 29.4	75.1 ± 42.4	0.33
Hemoglobin (g/L)	108.24 ± 13.90	112.92 ± 14.60	0.09
Albumin (g/L)	41.50 ± 1.98	41.33 ± 2.40	0.69
Glutamic-pyruvic transaminase (U/L)	14.0 ± 8.8	14.3 ± 11.7	0.88
Pre-dialysis BUN (mmol/L)	27.01 ± 3.85	26.59 ± 4.40	0.59
Post-dialysis BUN (mmol/L)	8.84 ± 2.77	8.71 ± 2.40	0.79
Pre-dialysis Scr (umol/L)	1093.0 ± 235.6	1052.9 ± 248.6	0.36
Post-dialysis Scr (umol/L)	382.1 ± 130.9	386.2 ± 123.6	0.86
Kt/V	1.38 ± 0.27	1.35 ± 0.22	0.58
Pre-dialysis serum potassium (mmol/L)	5.07 ± 0.70	5.09 ± 0.82	0.86
Sodium (mmol/L)	134.10 ± 3.39	134.40 ± 3.23	0.64
Calcium (mmol/L)	2.37 ± 0.20	2.26 ± 0.19	0.85
Phosphorus (mmol/L)	2.14 ± 0.55	2.17 ± 0.58	0.76

Data are presented as mean ± SD.

### Plasma metabolomics analysis by CE-MS

**Figure 1:**
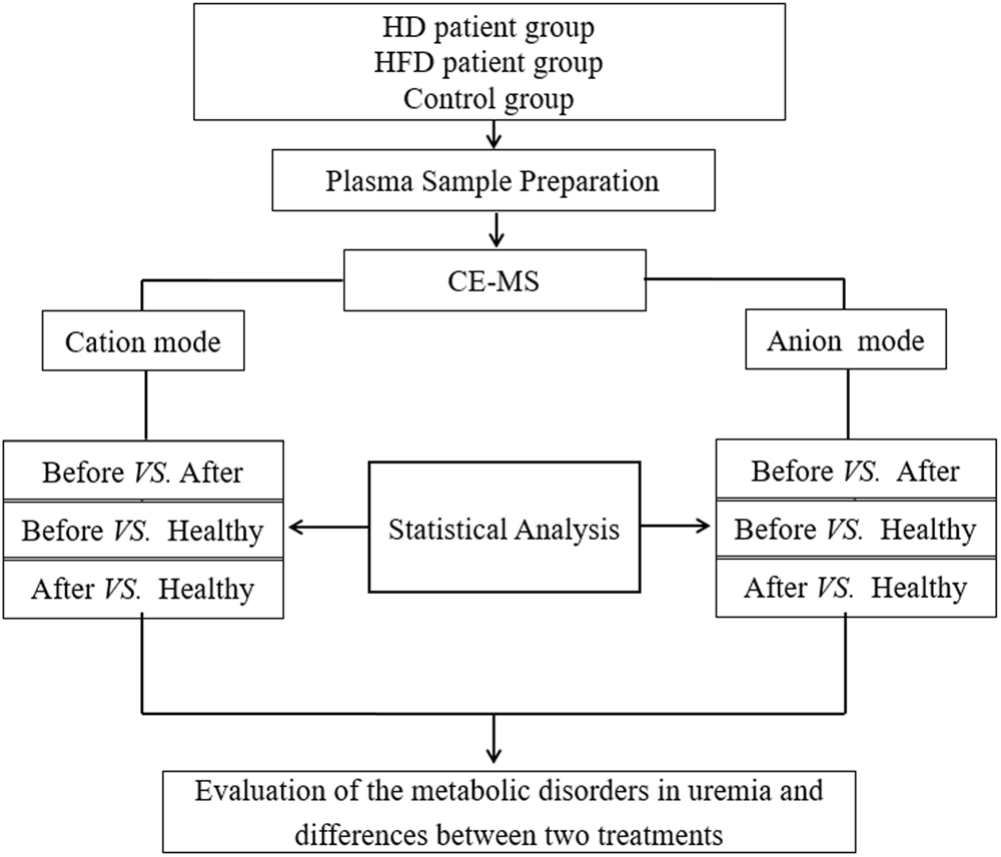
Flow chart used to display the whole process of the metabolomics study

Non-targeted metabolomics technology based on CE-TOF/MS was used to investigate plasma metabolites in HD patients, HFD patients and healthy controls. The whole workflow of this study is shown in Fig. 1. After qualitative analysis and the 80% rule used to exclude missing values^13^, a total of 228 metabolites were obtained. The RSD% distribution of IS-normalized data in 38 QC samples showed that 63.2% of compounds had a RSD% less than 30% and the intensity sum of them reached 98.1% of the total compounds. These results indicated that the metabolic profiling data in the current study were reliable. In the end, 144 compounds were subjected to subsequent statistical analysis.

**Figure 2:**
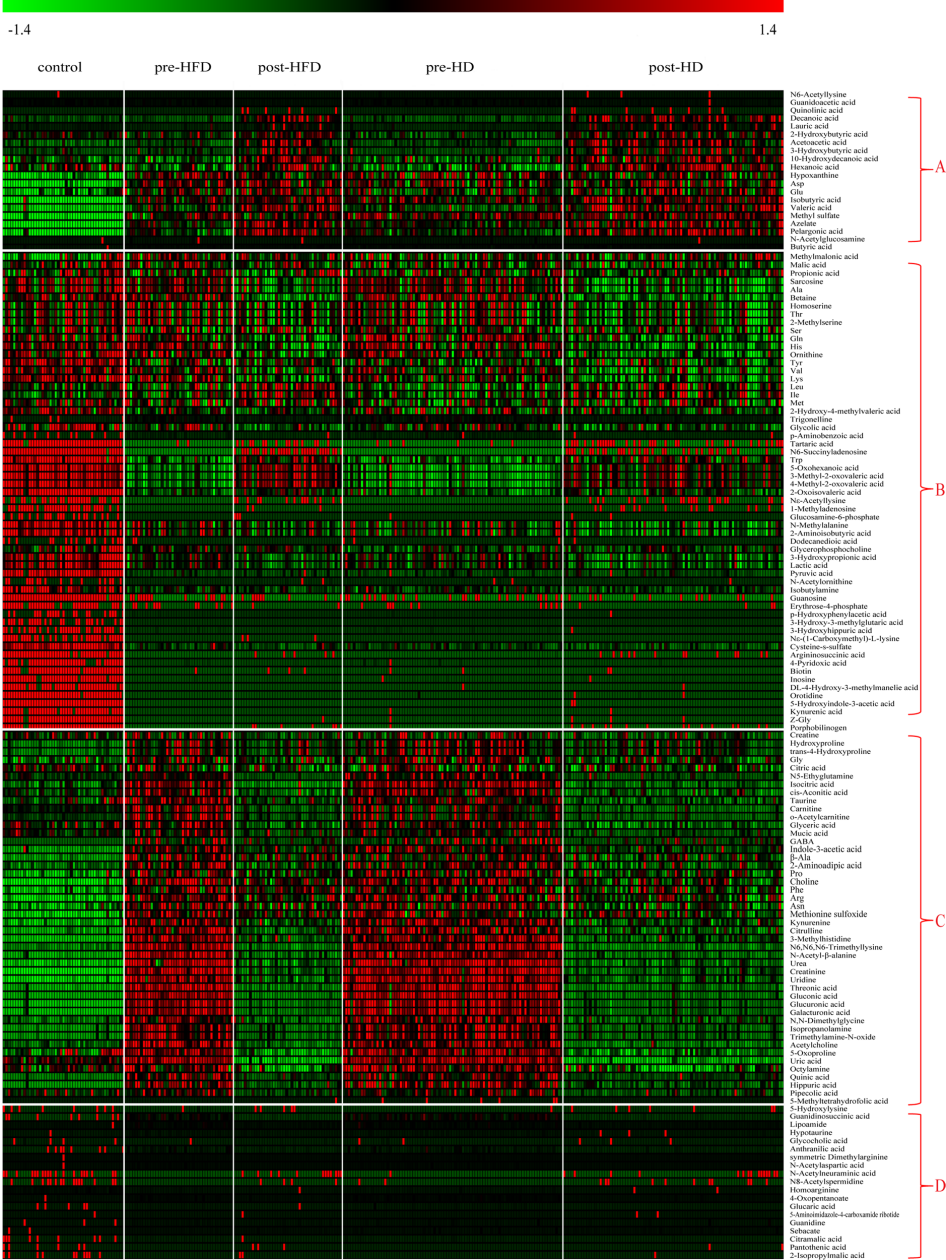
Heat maps of 144 plasma metabolites in five groups. Red, green and black indicate high, low and unchanged levels of metabolites, respectively.

First, the data from all samples were imported into SIMICA-P software for principal component analysis (PCA). R2X and Q2 of the PCA model were 0.53 and 0.45, respectively. Three distinct clustering patterns from the pre-treatment groups (including pre-HFD and pre-HD), the post-treatment groups (including post-HFD and post-HD), and the control group were located along the PC1 direction in the PCA score plot, which indicates that differences exist among study groups. However, two pre-treatment/post-treatment groups overlapped substantially with each other (see Supplementary Fig. S1). Second, the heat map was prepared with the MeV software to compare the relative changes in metabolite contents among different groups. According to the change tendency, the heat map could be delimited in four regions (Fig. 2). In region A, metabolites increased after dialysis compared to pre-dialysis, but they were very low in controls. In region B, the levels of compounds in controls were high but lower in patients no matter whether the samples were from pre-dialysis or post-dialysis. In region C, there was a strong accumulation of metabolites in the two pre-dialysis groups that almost decreased to normal levels after dialysis treatments. Notably, most metabolites in region C were uremic toxins. The results also indicated that the uremic toxins could be efficiently cleared by dialysis. In region D, the levels of the compounds were relatively stable among the 5 groups.

### Metabolic differences between uremic patients with maintaining dialysis and healthy controls

**Figure 3:**
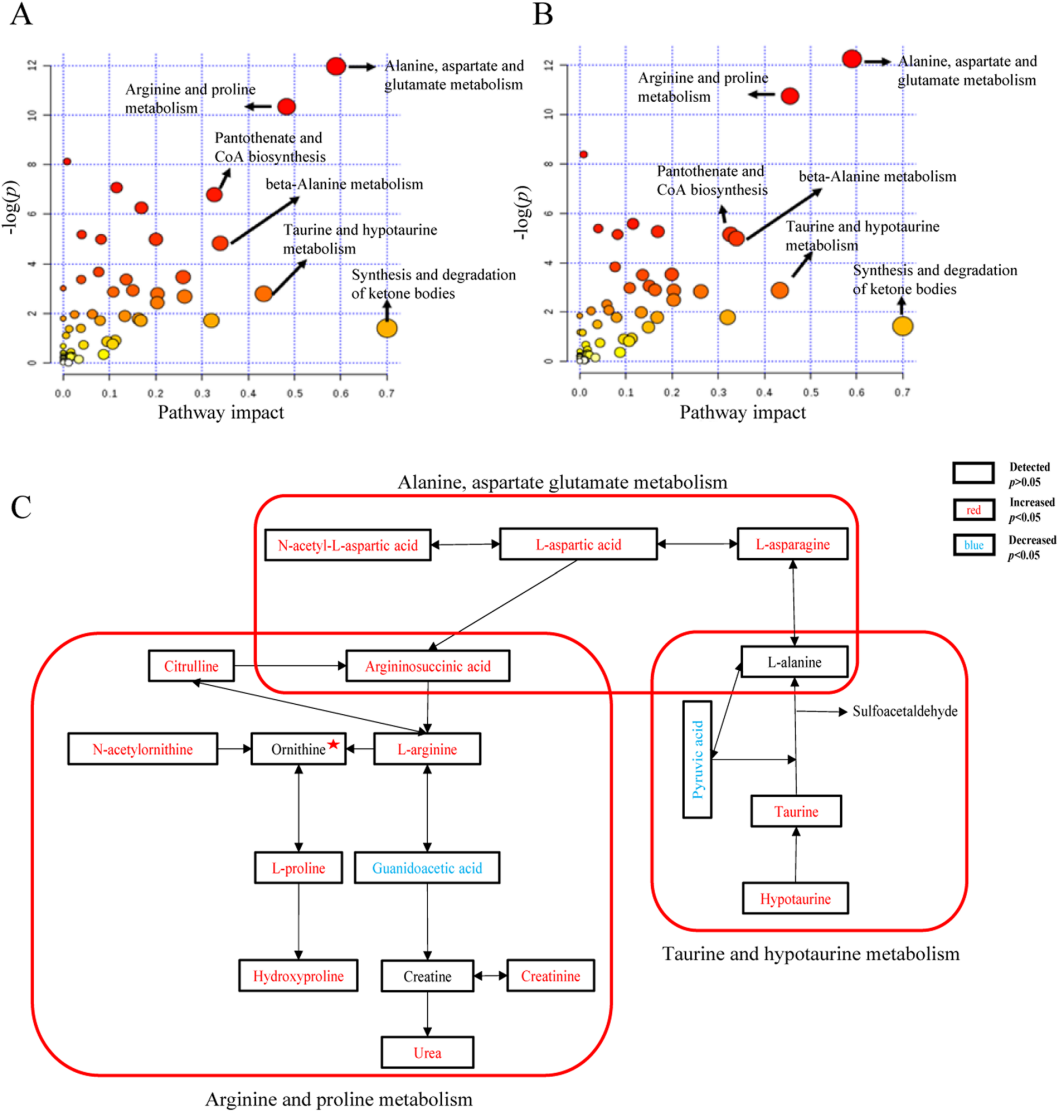
Metabolic comparison between patients and controls. (**A**) Changed pathways in uremia before treatments in pre-HD patients. (**B**) Changed pathways in uremia before treatments in pre-HFD patients. (**C**) Detailed changes of 18 metabolites in three important metabolic pathways when pre-dialysis patients are compared to controls. Metabolites marked with a red pentagram represent no differences between pre-HFD and the controls but are significantly reduced in pre-HD vs. the controls.

The selection of features was obtained by SVM-RFE. The number of metabolites and accuracy rates are displayed in Supplementary Table S1. A nonparametric test was then performed to further show all significantly changed metabolites in uremia with *p* < 0.05 followed by confirmation with FDR < 0.05. Significantly altered metabolites involving metabolic pathways were analyzed by MetaboAnalyst 3.0 (http://www.metaboanalyst.ca/). The results demonstrated that uremic patients undergoing HFD or HD treatments had similar metabolic alterations in alanine, aspartate and glutamate metabolism, arginine and proline metabolism, taurine and hypotaurine metabolism and so on (Fig. 3A,B). In total, 18 metabolites associated with three metabolic pathways (confirmed in the KEGG pathway database (www.genome.jp/kegg/)) were detected in this study. The close interrelations between these molecules are exhibited in Fig. 3C. It can be seen that most compounds had higher relative concentrations in patients compared to the controls, except for L-alanine, creatine, guanidoacetic acid, ornithine and pyruvic acid. Detailed information about these molecules is listed in Table 2. Taking urea as an example, its relative intensity increased in the pre-dialysis groups versus controls (17.30 ± 3.68 in pre-HFD, FC = 3.74 and 17.90 ± 3.28 in pre-HD, FC = 3.86 vs. 4.63 ± 1.12 in control) and then dramatically decreased to 7.87 ± 2.36 in post-HFD, FC = 1.70 and 7.66 ± 1.92 in post-HD, FC = 1.66. Significant differences in urea existed between pre-HFD and control (FDR < 0.001), between pre-HD and control (FDR < 0.001), between post-HFD and control (FDR < 0.001), and between post-HD and control (FDR < 0.001). Other compounds, including arginine (pre-HFD vs. control FC = 2.39, FDR < 0.001; pre-HD vs. control FC = 2.34, FDR < 0.001), argininosuccinic acid (7.12, <0.001; 7.60, <0.001), asparagine (1.44, <0.001; 1.44, <0.001), aspartic acid (2.42, <0.001; 2.24, <0.001), citrulline (3.56, <0.001; 3.40, <0.001), creatinine (14.52, <0.001; 14.15, <0.001), hydroxyproline (3.70, <0.001; 3.76, <0.001), hypotaurine (2.92, <0.001; 2.66, <0.001), N-acetylaspartic acid (4.75, <0.001; 4.39, <0.001), N-acetylornithine (5.63, <0.001; 4.74, <0.001), proline (1.73, <0.001; 1.78, <0.001), and taurine (1.88, <0.001; 1.36, <0.001), had significantly higher levels in pre-dialysis groups than in the control group. Whereas alanine (pre-HFD vs. control FC = 0.97, FDR = 0.541; pre-HD vs. control FC = 0.97, FDR = 0.298) and creatine (1.29, 0.651; 1.21, 0.730) showed no difference in pre-dialysis vs. control. Ornithine (0.95, 0.484; 0.89, 0.034) showed a difference only in pre-HD vs. control. The levels of guanidoacetic acid (pre-HFD vs. control FC = 0.82, FDR = 0.005; pre-HD vs. control FC = 0.81, FDR <0.001) and pyruvic acid (0.09, <0.001; 0.10, <0.001) were significantly lower in the pre-dialysis groups vs. the controls. Furthermore, alanine (post-HFD vs. control FC = 0.77, FDR <0.001; post-HD vs. control FC = 0.71, FDR < 0.001), arginine (2.06, <0.001; 1.93, <0.001), argininosuccinic acid (1.36, <0.001; 1.75, <0.001), asparagine (1.27, <0.001; 1.19, <0.001), citrulline (1.60, <0.001; 1.67, <0.001), creatine (0.75, 0.004; 0.78, 0.002), creatinine (5.37, <0.001; 5.31, <0.001), guanidoacetic acid (0.35, <0.001; 0.37, <0.001), hydroxyproline (2.27, <0.001; 2.31, <0.001), hypotaurine (1.60, <0.001; 1.46, <0.001), N-acetylaspartic acid (1.93, <0.001; 2.09, <0.001), ornithine (0.70, <0.001; 0.65, <0.001), proline (1.41, <0.001; 1.38, <0.001), taurine (0.92, 0.941; 0.74, 0.014), and urea (1.70, <0.001; 1.66, <0.001) prominently declined after dialysis treatments. Only aspartic acid (post-HFD vs. control FC = 2.52, FDR <0.001; post-HD vs. control FC = 2.31, FDR <0.001), N-acetylornithine (6.04, <0.001; 4.10, <0.001), and pyruvic acid (0.12, <0.001; 0.11, <0.001) showed no significant differences after dialysis. The results indicated that dialysis treatments can efficiently decrease most metabolites in these pathways.

**Table 2:** Alterations of relative levels of 18 important metabolites in three pathways.

Metabolites	FC (pre-HFD vs. ctrl)	FDR (pre-HFD vs. ctrl)	FC (post-HFD vs. ctrl)	FDR (post-HFD vs. ctrl)	FC (pre-HD vs. ctrl)	FDR (pre-HD vs. ctrl)	FC (post-HD vs. ctrl)	FDR (post-HD vs.ctrl)
Alanine	0.97	0.541	0.77	<0.001	0.97	0.298	0.71	<0.001
Arginine	2.39	<0.001	2.06	<0.001	2.34	<0.001	1.93	<0.001
Argininosuccinic acid	7.12	<0.001	1.36	<0.001	7.60	<0.001	1.75	<0.001
Asparagines	1.44	<0.001	1.27	<0.001	1.44	<0.001	1.19	<0.001
Aspartic acid	2.42	<0.001	2.52	<0.001	2.24	<0.001	2.31	<0.001
Citrulline	3.56	<0.001	1.60	<0.001	3.40	<0.001	1.67	<0.001
Creatine	1.29	0.651	0.75	0.004	1.21	0.730	0.78	0.002
Creatinine	14.52	<0.001	5.37	<0.001	14.15	<0.001	5.31	<0.001
Guanidoacetic acid	0.82	0.005	0.35	<0.001	0.81	<0.001	0.37	<0.001
Hydroxyproline	3.70	<0.001	2.27	<0.001	3.76	<0.001	2.31	<0.001
Hypotaurine	2.92	<0.001	1.60	<0.001	2.66	<0.001	1.46	<0.001
N-Acetylaspartic acid	4.75	<0.001	1.93	<0.001	4.39	<0.001	2.09	<0.001
N-Acetylornithine	5.63	<0.001	6.04	<0.001	4.74	<0.001	4.10	<0.001
Ornithine	0.95	0.484	0.70	<0.001	0.89	0.034	0.65	<0.001
Proline	1.73	<0.001	1.41	<0.001	1.78	<0.001	1.38	<0.001
Pyruvic acid	0.09	<0.001	0.12	<0.001	0.10	<0.001	0.11	<0.001
Taurine	1.88	<0.001	0.92	0.941	1.36	<0.001	0.74	0.014
Urea	3.74	<0.001	1.70	<0.001	3.86	<0.001	1.66	<0.001

**Figure 4:**
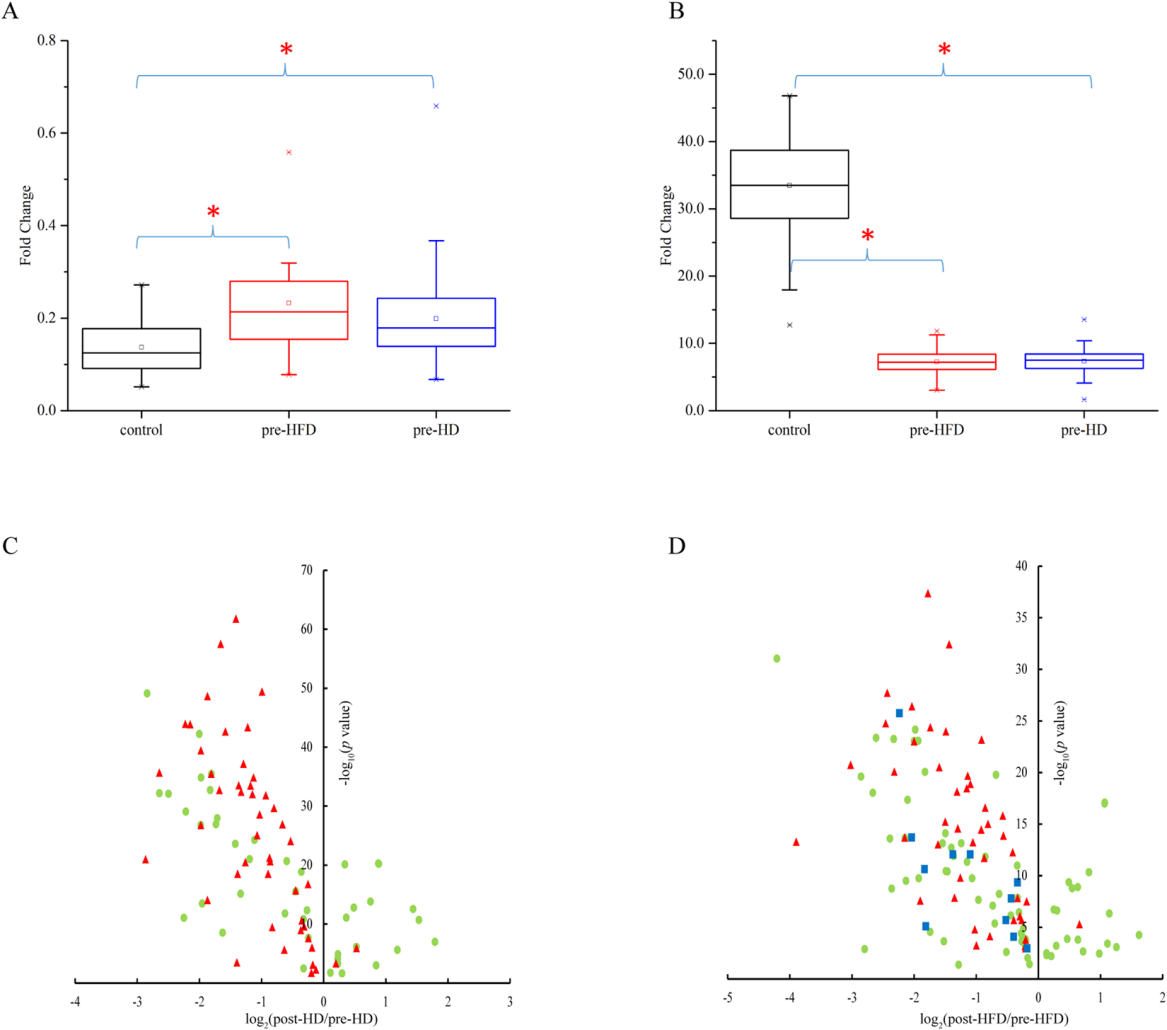
Significant changes in metabolites. (**A**) Box plot of Glu/Gln in control, pre-HFD and pre-HD groups. (**B**) Box plot of Trp/Kyn in control, pre-HFD and pre-HD groups. (**C**) Volcano plot of significantly altered metabolites (*p* < 0.05) in HD treatment. (**D**) Volcano plot of significantly altered metabolites (*p* < 0.05) in HFD treatment. Red triangle indicates uremic retention solutes and blue box shows additionally reduced uremic retention solutes in HFD treatment.

The ratio of glutamate/glutamine (Glu/Gln) and tryptophan/kynurenine (Trp/Kyn) have been reported to have close relationships with enzymes. In this experiment, the values of Glu/Gln (i.e., 0.23 ± 0.10 in pre-HFD, FDR <0.001; 0.20 ± 0.09 in pre-HD, FDR <0.001 vs. 0.14 ± 0.06 in control) in two pre-dialysis groups were significantly increased compared with the values in the controls (Fig. 4A,B). Trp/Kyn was 7.24 ± 1.87 (FDR < 0.001 vs. control) in pre-HFD, 7.35 ± 1.85 (FDR < 0.001 vs. control) in pre-HD and 33.45 ± 7.59 in control. Neither Glu/Gln (FDR = 0.07) nor Trp/Kyn (FDR = 0.806) showed any difference between pre-HFD and pre-HD.

### Metabolite differences between HD and HFD treatments

The classification performance of pre-HD vs. post-HD obtained by SVM-RFE was 99.28% ± 0.52%, and 99 individual molecules remained for further statistical analysis. The classification performance and the number of selected individual molecules were 100.00 ± 0.00% and 144 in the case of pre-HFD vs. post-HFD. Differential metabolites of HD treatment and HFD treatment were respectively investigated by a paired T test in SPSS software. The volcano plots of the significantly changed metabolites in HD and HFD are presented in Fig. 4C,D. In total, 91 individual molecular species revealed significant differences (*p* < 0.05, FDR < 0.05) between pre-HD and post-HD groups, and 47 of them were uremic retention solutes (Fig. 4C). Additionally, 126 individual molecules, in which 55 were uremic retention solutes, showed significant differences (*p* < 0.05, FDR < 0.05) between pre-HFD and post-HFD groups (Fig. 4D). Among them, 44 uremic retention solutes had common trends (43 decreased and 1 increased). The volcano plots demonstrated that most metabolites presented a trend of decline after HD or HFD treatment. Moreover, 45 uremic retention solutes significantly decreased after HD therapy and 54 uremic retention solutes substantially decreased after HFD. Generally, uremic toxins could be efficiently removed by both dialysis methods. Specifically, 11 uremic retention solutes (i.e., guanidine, hippuric acid, cis-aconitic acid, quinic acid, citramalic acid, asparagine, ornithine, methionine sulfoxide, choline, trigonelline, orotidine) were efficiently cleared by HFD rather than HD. The majority of them played an important role in many adverse effects or even nephrotic syndrome^14, 15^.

## Discussion

Plasma metabolism in patients with ESRD has been reported to change in response to dialysis therapy. However, dialysis-pattern-dependent changes in plasma metabolites remain poorly understood. To this end, metabolic differences in the plasma of uremic patients receiving HD and HFD treatments were thoroughly investigated in this study. Our findings showed that a large number of common uremic retention solutes were cleared after both therapies. Interestingly, 11 uremic retention solutes were much more efficiently cleared by HFD than by HD. Typical uremic retention solutes uniquely cleared by HFD rather than HD were guanidine, hippuric acid, amino acids and alkalines.

Guanidine is a small, water-soluble solute. De Deyn *et al*. reported that guanidine was highly accumulated in kidney failure patients^16^. It can exhibit neurotoxic influence^17^, a pro-inflammatory effect on leukocytes^15^, a potential damaging effect on blood vessels^15^ and structural modification effects on albumin by deamidation^18^. Additionally, intradialytic kinetic analysis of guanidine showed that guanidine’s distribution volume was significantly higher than urea, so it was much more difficult to be cleared during the hemodialysis regimen^19^. The more efficient clearance of guanidine in HFD than in HD in the present study may account for the better clinical treatment effects of HFD because it led to patients having a better quality of life with fewer complications than HD.

Organic acids always show elevated levels in uremia. As a typical accumulated uremic toxin, hippuric acid has been characterized as a difficulty that could be cleared by a dialysis regimen^3^. Multidrug resistance protein 4 (MRP4) and breast cancer resistance protein (BCRP) are two significant efflux pumps in nephridium to transport wastes over the apical membrane to urine^20, 21^, but MRP4- and BCRP-mediated transports can be inhibited by hippuric acid^22^. Hippuric acid can also contend with other organic acids for the secretory pathway in the proximal nephron^23^ so that metabolic acidosis in kidney disease is severely aggravated^14^. Additionally, another disadvantage of the high level of hippuric acid is its inhibition of glucose utilization in the muscles^24^. Therefore, the accumulation of hippuric acid may account for the progression of muscular weakness in uremic patients. Overall, it is necessary to pay more attention to reductions in hippuric acid in developing treatments for CKD because its accumulation can directly cause less excretion of other acid compounds from the kidneys into urine. The better efficiency of removing hippuric acid from blood by HFD than by HD may provide another support for HFD to lead to a better clinical efficacy.

In addition, there were some disparities in the removal efficiencies of HFD and HD in some amino acids and alkaline compounds. Although amino acids have been detected in CKD patients, to the best of our knowledge, details of their influence on uremic complications are unclear. Methionine sulfoxide is an oxidative product. It may be produced from methionine oxidation by reactive oxygen species. In this study, significantly increased methionine sulfoxide was found in uremic patients compared to the controls. This finding may partially support the increased oxidative stress that occurs in kidney disease^25^. Furthermore, accumulation of choline was reported to have a close association with renal insufficiency^26^. Notably, this increased concentration of choline may reflect the improved protein intake according to guidelines for dialysis patients^27^. However, the negative influence of accumulated plasma choline has not been investigated in detail. It would be a good idea to analyze the effects of cumulative choline in cells or organs for improving our understanding of the pathology of uremia.

Our findings also illustrated that both HFD and HD led to alterations in arginine and proline metabolism as well as taurine and hypotaurine metabolism. Taking guanidoacetic acid as an example, there was a significantly lower level in patients than in controls, which was consistent with a previous report^28^. Moreover, urea and creatinine significantly accumulated in patients compared to controls, which confirmed the pathological condition of uremic patients. The elevated urea and creatinine could be caused by reduced renal function. However, the mechanism for guanidoacetic acid to first metabolize to creatine and then metabolize to urea and creatinine might be distinctly enhanced by considerations of reduced guanidoacetic acid, and there was no prominently changed creatine but increased creatinine and urea levels. It might be rational to speculate that the increased creatinine level in CKD was associated with increased energy consumption/waste, which was a possible explanation for the clinical symptoms of emaciation and muscle weakness in uremia. Increased urea also had many adverse influences on complications because it was closely related to carbamylation *in vivo* ^29^. Our results also showed higher levels of arginine and citrulline in uremia. Arginine was important to maintain the stability of the internal environment. Though renal function was decreased in uremia, arginine synthesis was still preserved^30^. Arginine as an acidifying agent might alter the acid-base equilibrium in CKD, and increased arginine may promote kidney injury^31^. Because metabolic acidosis was regarded as an important reason for body mass wasting in patients^32^, it might be reasonable to consider arginine as another possible factor for inducing emaciation of uremic patients.

Furthermore, the elevated levels of hypotaurine and taurine detected in the plasma of uremic patients were consistent with a previous report^33^. Hypotaurine is considered to play a protective role for scavenging free radicals caused by oxygen and biological antioxidants^34^. The increased hypotaurine and taurine in this study have occurred and it can be speculated that uremic patients have many more free-radicals due to increased oxidative stress. To maintain homeostasis, more hypotaurine was needed to form taurine. However, excessive accumulation of extra- or intracellular taurine could cause dizziness in ESRD patients^35^. In our research, hypotaurine and taurine obviously decreased after dialysis. Generally, most of the metabolites in the pathways discussed above showed a significant decline after dialysis treatment, except for aspartic acid, N-acetylornithine and pyruvic acid.

Finally, two important ratios (i.e., Glu/Gln and Trp/Kyn) that are respectively associated with the enzymes of glutaminase and indoleamine 2,3-dioxygenase (IDO) were compared among the study groups. Glutaminase plays a critical role in regulating the level of glutamate in humans by converting glutamine to glutamate in cells. In this study, Glu/Gln was significantly higher in pre-HD and pre-HFD samples than in the controls (Fig. 4A), which implied that there was increased glutaminase activity in uremic patients. Because glutamate was reported to be an excitatory neurotransmitter in central nervous system disorders^36^, it might be argued that increased Glu/Gln in uremic patients has a close relationship with nervous system disorders and energy metabolism in uremia. Additionally, inhibiting glutaminase activity or decreasing Glu/Gln may be a new idea for improving quality of life for uremic patients. Tryptophan is an essential amino acid and can be degraded to kynurenine by IDO. Our findings revealed that individual tryptophan and the ratio of Trp/Kyn were much lower in pre-HD and pre-HFD than in the controls, whereas the individual kynurenine was prominently higher in patients (Fig. 4B). Such results were consistent with the previous study in which IDO was reported to increase substantially in CKD^37^. There is a certain possibility that the consumption of tryptophan in human body induces inactivation or enervation of T cells and even its apoptosis so that the microenvironment of immunosuppression is eventually generated^38^. Moreover, kynurenine is an endogenous ligand of the human aryl hydrocarbon receptor (AHR) and plays an important role in suppressing immune responses^39, 40^. Furthermore, it has close links with carotid atherosclerosis in ESRD^9^. Overall, it seems reasonable to conjecture that suppressed immune responses and increased atherosclerosis risk in uremic patients may be partially caused by increased activity of AHR and accumulated kynurenine.

In conclusion, a CE-MS-based metabolomics profile was successfully applied to investigate dialysis-dependent metabolic changes in uremic patients with the multivariate analysis method SVM-RFE and a univariate analysis. Pronounced metabolite changes were found between pre-dialysis and post-dialysis for both HFD and HD therapies. Eleven uremic retention solutes were cleared better in HFD mode, which may partially explain from a metabolomics aspect why HFD has better therapeutic efficacies on uremia than HD. Meanwhile, our findings also supported the point that maintaining both HFD and HD therapies shows beneficial effects on uremia by efficiently removing a large number of uremic retention solutes. Our results demonstrated that a CE-MS-based metabolomics approach has potential power in providing in-depth knowledge for understanding uremic metabolic alterations and the effects of different dialysis types on uremia and could even provide future guidance for personalized dialysis therapy.

## Methods

### Study population

Patients who were 18 to 80 years old who were undergoing high-flux hemodialysis (HFD) or low-flux hemodialysis (HD) in the hemodialysis center of Dalian Municipal Central Hospital three times a week and who had been undergoing hemodialysis for three or more months were enrolled. This study was reviewed and approved by the ethics committee of Dalian Municipal Central Hospital (Dalian, China). The experiment was performed according to the approved guidelines. All the participants voluntarily provided informed written consent after reviewing a written plan of the whole study. In total, there were 57 maintenance HFD patients and 557 HD patients in this center by Dec 2014. To address the concerns that the metabolomics results were probably influenced by large heterogeneity in baseline clinical characteristics between HFD and HD patients, we performed propensity score matching (HFD: HD = 1:2), which incorporated and adjusted all the following baseline clinical differences between HFD and HD patients, including age, gender, primary disease, dialysis age, hemoglobin, albumin, glutamic-pyruvic transaminase, pre/post-dialysis urea, serum creatinine, Kt/V, pre-dialysis serum potassium, sodium, calcium, and phosphorus. Plasma samples of patients were collected at the start of HFD/HD and at the end of HFD/HD. Samples of healthy controls were collected in the Dalian Physical Examination Center. Only subjects without chronic respiratory disease, cardiovascular disease, diabetes mellitus, gastroenteric disease, or severe renal or hepatic disease were selected as the healthy controls. All the plasma samples were stored at −80 °C until sample preparation.

### Materials and Chemicals

The dialyzer with low flux used in this study was the Fresenius F6, and the dialyzer with high flux was the Fresenius F60. Liquid chromatography grade methanol and chloroform were purchased from Merck (Darmstadt, Germany). Liquid chromatography grade ammonium acetate was purchased from Sigma-Aldrich (St. Louis, Mo, USA), and 98% formic acid was purchased from Fluka (Darmstadt, Germany). Ultrapure Water (18.2 MΩ-cm, TOC = 6 ppb) was prepared with a Milli-Q system (Millipore, Billerica, MA, USA). 10 mM D-camphor-10-sulfonic acid sodium salt (Wako, Japan) and 10 mM L-methionine sulfone (Wako, Japan) in methanol were used as internal standard solution 1 (ISS1). A mixture of 1 mM disodium 3-hydroxynaphthalene-2,7-disulfonate (Wako, Japan), trimesic acid (Wako, Japan), N,N-diethyl-2-phenylacetamide (Wako, Japan), and 3-aminopyrrolidine dihydrochloride (Aldrich, USA) in water was used as internal standard solution 3 (ISS3). ISS1 was used to standardize the metabolite intensity and ISS3 was used to adjust the migration time of the metabolites.

### Sample preparation

Plasma samples were first thawed on ice, and 50 μL of plasma was mixed with 450 μL of ice-cold ISS1 in a 1.5-mL Eppendorf tube. After thoroughly vortexing the samples for 60 sec, 500 μL of chloroform was added, and then the mixture was vortexed at room temperature for 60 sec. A two-phase system was formed by adding 200 μL of Milli-Q water followed by another 30 sec of vortexing. The obtained mixture was settled at 4 °C for 10 min. After centrifugation at 14000 rpm for 15 min at 4 °C, the supernatant was respectively transferred to a Millipore 5-kDa ultrafiltration membrane (200 μL for negative mode and 270 μL for positive mode) to remove latent impurities (e.g., proteins) by centrifuging at 11000 rpm for 2 hours at 4 °C. Then, the filtered fluid was freeze-dried in CentriVap Centrifugal Vacuum Concentrators (Labconco, MO). Twenty microliters of ISS3 was added to 200 μL freeze-dried residue as a “negative” reconstitution solution. Eighty microliters ISS3 was added to 270 μL freeze-dried residue as a “positive” reconstitution solution. After being vortexed for 30 sec and centrifuged at 14000 rpm for 10 min at 4 °C, 10 μL of supernatant was transferred into an injection vial for CE-TOF/MS analysis. Notably, pooled quality control (QC) samples were obtained by pipetting an equal volume of plasma from each sample, and the QCs were pretreated in the same manner as the previous biological samples. QC samples were applied to appraise the reliability of the whole experimental process, including sample preparation and CE-TOF/MS sequence running.

### CE-TOF/MS-based plasma metabolomics profiling

Plasma metabolomics analysis was analyzed by capillary electrophoresis (G7100A, Agilent, USA) coupled with time of flight mass spectrometry (G6224A, Agilent, USA). The CE system was controlled by ChemStation software (B.04.03, Agilent), and the TOF/MS system was controlled by Mass Hunter Workstation software (B.04.00, Agilent). During the whole analytical process, the sample tray temperature was maintained below 5 °C through a minichiller (Huber, Germany). The metabolites of each reconstituted sample were separated on a fused-silica capillary (80 cm × i.d. 50 μm) (Human Metabolome Technologies (HMT), Inc., Japan), and the temperature of the capillary was maintained at 20 °C. A methanol/water solution (1:1, V/V) containing 0.1 μM hexakis (2,2-difluoroethoxy) phosphazene was used as a sheath liquid and a coaxial sheath liquid interface was developed to realize coupling of CE with TOF/MS.

Data acquisition was performed in electrospray ionization (ESI) cation and anion full scan modes. In positive mode, the capillary voltage was 27 kV, and each sample ran for 30 minutes. Water containing 3.95% formic acid (PH = 1.8) was used as the background electrolyte, and after 10 injections, it was renewed. The sample solution was injected for 3 sec at 50 mbar (i.e., 3 nL). In negative mode, the capillary voltage was 30 kV, and each sample ran for 40 minutes. An extra 15 mbar internal pressure was applied to the inlet capillary to facilitate the electroosmotic flow. The fused silica capillary was first preconditioned with an electrolyte containing phosphate (H3302-1022, HMT, Japan) and washed with the running electrolyte (50 mM ammonium acetate). The electrolyte was renewed after each injection with the help of a buffer replenishment system. Sample solution was injected for 25 s at 50 mbar (i.e., 25 nL). The detailed mass parameters were as follows: nebulizer pressure was set at 5 psig; dry gas temperature was set at 300 °C; nitrogen flow was set at 7 L/min; skimmer was set at 50 V; Oct RFV was set at 650 V; capillary voltage was set at 4 kV (+) and 3.5 kV (−); the fragmentor was set at 105 V (+) and 125 V (−); and mass ranges were 60–1000 (+) and 50–1000 (−), respectively. The real-time calibration of the exact mass measurement was achieved using the reference mass provided by the protonated isotope.

### Data analysis

The raw data from CE-TOF/MS were first imported in a qualitative software (B.04.00, Agilent) to extract the migration time of internal standards. Through the software of MethodMarker (HMT, Japan), the real migration time in the experiment was corrected to standard migration time so that it could be used for further peak identification based on a database that consists of 507 metabolite standards (259 positive, 248 negative) provided by HMT (Japan). Quantitative Analysis Software (B.04.00, Agilent) was used to extract and identify peak ions according to accurate masses and corrected migration time. The mass accuracy was set ±20 ppm and the migration time window was set ±1.5 min for the quantitative analysis. After normalizing the data by IS, the relative standard deviation (RSD) of detected metabolites in QCs was calculated to evaluate the stability of the whole experiment. Only metabolites with RSD less than 30% were used for subsequent statistical analyses.

SVM-RFE is a recursive feature selection algorithm that starts from all the input features. In each iteration, the SVM model is constructed based on the current feature subset and the weight of each feature is calculated based on the contribution to the hyper-plane. Features with the lowest weights are removed from the current feature subset. The procedure is continued until the current feature subset is empty. When the iteration terminates, the feature subset with the best performance is kept as the selected feature subset. To investigate different alterations of metabolites after HFD and HD treatments and to study the related metabolic deregulation in uremic patients to explore a disease mechanism, we divided the studied subjects into six sub-groups: pre-HD vs. post-HD, pre-HFD vs. post-HFD, control vs. pre-HD, control vs. pre-HFD, control vs. post-HD, and control vs. post-HFD. In this study, the kernel function of SVM was linear, and 5% of features with the lowest weights were removed in each iteration. The implementation of SVM can be accessed at http://www.csie.ntu.Edu.tw/∼cjlin/libsvm. Unit variance (UV) scaling was used to put the variables on a comparable scale. As threefold cross-validation was run 100 times, the features whose selected frequencies were larger than or equal to 0.8 remained for each sub-group.

The data were handled with SIMCA-P software (version 11.0; Umetrics) to acquire principal component analysis (PCA) with UV scaling in 5 groups. Additionally, SPSS 18 (SPSS Inc., Chicago, USA) was used to perform the nonparametric Mann-Whitney U test. False discovery rate (FDR) was all measured by Matlab software (The MathWorks, USA) with the Benjamini and Hochberg method. All the statistical tests were applied to evaluate the differences among groups, and if *p* value < 0.05 with an FDR limit equal to 0.05, significant differences are supposed to exist.

## Electronic supplementary material

Supplementary information
