# Supplementary material for: Plasma metabolomics profiling of maintenance hemodialysis based on capillary electrophoresis - time of flight mass spectrometry
Source: Sci Rep. 2017 Aug 15;7:8150. doi: 10.1038/s41598-017-08327-w (PMC5557835; doi:10.1038/s41598-017-08327-w)
Supplement: Supplementary file 1 — Supplementary information [file 41598_2017_8327_MOESM1_ESM.pdf]

Plasma metabolomics profiling of maintenance hemodialysis based on capillary electrophoresis - time of flight mass spectrometry

Shuxin Liu[1#], Lichao Wang[2,3,5,#], Chunxiu Hu[2,5*], Xin Huang[4], Hong Liu[1], Qiuhui Xuan[2,5], Xiaohui Lin[4], Xiaojun Peng[3], Xin Lu[2,5], Ming Chang[1*], Guowang Xu[2,5*]

[1] Nephrology Department, Dalian Municipal Central Hospital, 826 Xinan Road, Dalian 116033, China.

[2] CAS Key Laboratory of Separation Sciences for Analytical Chemistry, Dalian Institute of Chemical Physics, Chinese Academy of Sciences, 457 Zhongshan Road, Dalian 116023, China.

[3]State Key Laboratory of Fine Chemicals, Dalian University of Technology, Dalian 116023, China.

[4]School of Computer Science & Technology, Dalian University of Technology, 116024, Dalian, China.

[5] University of Chinese Academy of Sciences, Beijing 100049, China.

[#] These authors contributed equally to this work. 1 and 2 have the equal intelligent knowledge right.

Supplementary Information

Table S1. No. of metabolites and accuracy rates obtained from SVM-RFE

Data	No. (frequency ≥ 80%)	SVM-RFE
pre-HD VS post-HD	99	99.28%±0.52%
pre-HFD VS post-HFD	144	100.00%±0.00%
pre-HD VS control	144	99.76%±0.37%
pre-HFD VS control	144	100.00%±0.00%
post-HD VS control	49	98.76%±0.71%
post-HFD VS control	70	97.86%±0.79%

Data are presented as mean ± SD

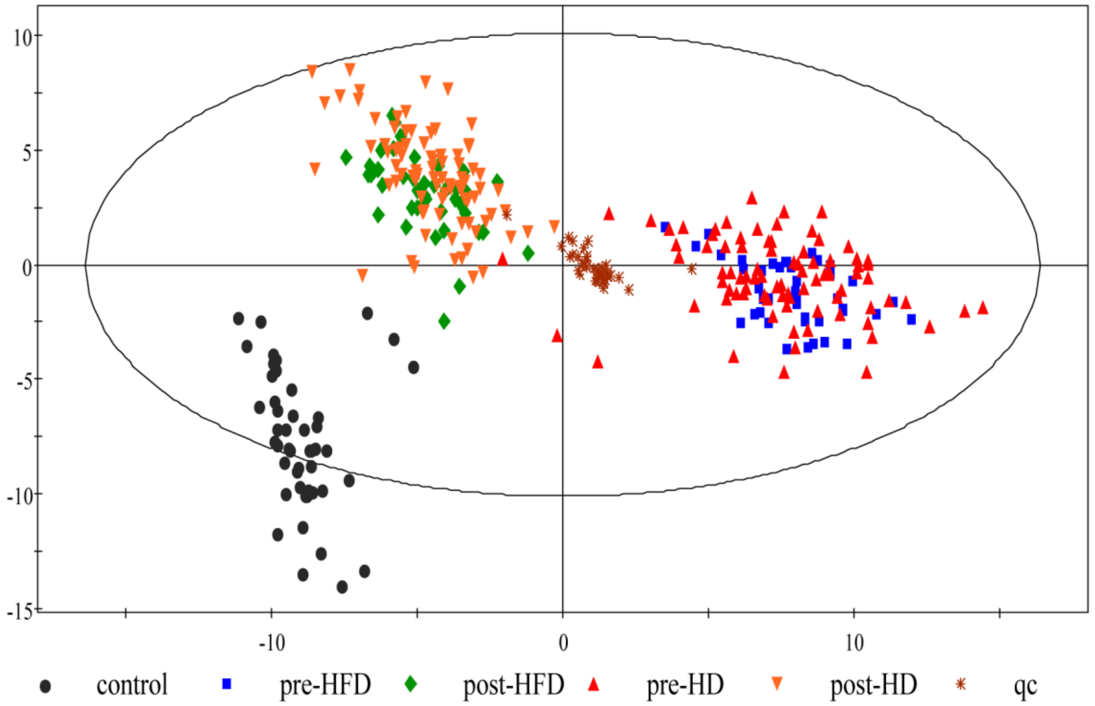

Figure S1 | PCA score plot of plasma metabolites in five groups after unit variance (UV) scaling pretreatment. Black dot means control group; blue box means pre-HFD group; green diamond means post-HFD group; red triangle means pre-HD group; orange inverted triangle means post-HD; brown star means qc group.
